# Nanofluid optical property characterization: towards efficient direct absorption solar collectors

**DOI:** 10.1186/1556-276X-6-225

**Published:** 2011-03-15

**Authors:** Robert A Taylor, Patrick E Phelan, Todd P Otanicar, Ronald Adrian, Ravi Prasher

**Affiliations:** 1 Arizona State University, Tempe, AZ, USA; 2 Loyola Marymount University, Los Angeles, CA, USA

## Abstract

Suspensions of nanoparticles (i.e., particles with diameters < 100 nm) in liquids, termed nanofluids, show remarkable thermal and optical property changes from the base liquid at low particle loadings. Recent studies also indicate that selected nanofluids may improve the efficiency of direct absorption solar thermal collectors. To determine the effectiveness of nanofluids in solar applications, their ability to convert light energy to thermal energy must be known. That is, their absorption of the solar spectrum must be established. Accordingly, this study compares model predictions to spectroscopic measurements of extinction coefficients over wavelengths that are important for solar energy (0.25 to 2.5 μm). A simple addition of the base fluid and nanoparticle extinction coefficients is applied as an approximation of the effective nanofluid extinction coefficient. Comparisons with measured extinction coefficients reveal that the approximation works well with water-based nanofluids containing graphite nanoparticles but less well with metallic nanoparticles and/or oil-based fluids. For the materials used in this study, over 95% of incoming sunlight can be absorbed (in a nanofluid thickness ≥10 cm) with extremely low nanoparticle volume fractions - less than 1 × 10^-5^, or 10 parts per million. Thus, nanofluids could be used to absorb sunlight with a negligible amount of viscosity and/or density (read: pumping power) increase.

## Introduction

Nanofluids, or suspensions of nanoparticles in liquids, have been studied for at least 15 years and have shown promise to enhance a wide range of liquid properties [1-20]. In the last few years, the co-authors [21-23] and others [24,25] have explored their potential towards developing a new type of direct absorption (or volumetric) solar thermal collector. The ideal volumetric thermal collector should: (1) efficiently absorb solar radiation (in the wavelength range - 0.25 <*λ *< 2.5 μm) and convert it to heat directly inside the working fluid, (2) minimize heat losses by convection and radiation (in the wavelength range - *λ *> 4 μm), and (3) keep system fouling/clogging and pumping costs to a minimum. The focus of this article is to explore condition (1) in detail for nanofluids.

As for (2) and (3), we believe that a nanofluid collector could meet these conditions as well. An effective way to address (2) is the use (possibly a few layers) of anti-reflective glazing as a cover to the solar collector. This cover would also need to be highly transparent to sunlight. With recent advances in low-e windows, solar collectors, and optical materials in general, there are several commercial glazing materials that meet these requirements - for examples, see [26,27]. For condition (3), one of the main promising factors of nano-sized particles is that as opposed to larger-sized particles, they can be put into conventional liquid pumping and plumbing with little adverse affects (i.e., without abrasion or clogging) [7,10]. Also, as will be discussed, ideal nanoparticle volume fractions end up being < 0.001 vol.% for sizable solar collector fluid depths. This means that incorporating nanoparticles in a system will not require much additional capital investment. Further, it is relatively easy to argue that the pumping power will not increase significantly for this level of particle volume fraction. To show this, the following equation for effective viscosity in a nanofluid [28] is used:(1)

where *μ*_eff _and *μ*_f _refer to the effective nanofluid viscosity and the base fluid viscosity, respectively. Also, *C*_μ _can be found through a relation to several other fluid parameters - see [28]. For many cases, though, *C*_μ _= 10 is a reasonable approximation [28]. If we plug in *f*_v _< 1 × 10^-5^, we can see that there is a negligible change in viscosity (i.e., *μ*_eff _≈ *μ*_f_). If viscosity is unchanged, it is even less likely that density would change at these low volume fractions. Thus, pumping power (for a *stable *nanofluid) will not change. For these reasons, nanofluids compare favorably with black dye and micro/macroparticle laden liquids. They are also expected to show enhancement over conventional surface-based collectors [21-25].

On the other hand, recent research indicates that nanofluids must be very carefully chosen to match their application in order to see enhancement. This is especially true for the nanofluid optical properties in a solar collector. If the volume fraction of nanoparticles is very high, all the incoming light will be absorbed in a thin surface layer where the thermal energy is easily lost to the environment. On the other hand, if the volume fraction of nanoparticles is low, the nanofluid will not absorb all the incoming solar radiation. Therefore, the optical properties of the fluid must be controlled very precisely or a nanofluid could actually be detrimental in a solar collector. This article first describes some simple modeling (using bulk properties) approaches that we used to explore how a nanofluid absorb sunlight. Next, we will describe our experimentation methods towards this same end. These results will then be compared and discussed. Lastly, this study presents some nanofluid recipes with cost estimates for solar collector applications.

## Modeling approach

**Figure 1:**
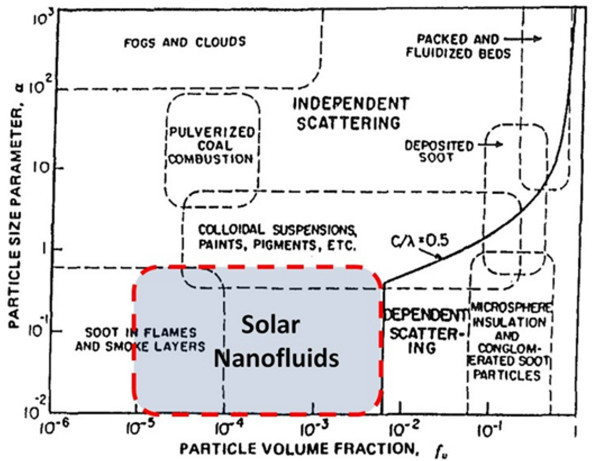
**Scattering regime map showing the boundary between dependent and independent scattering **[30].

In general, for cost-effective absorption, particles must be made from low-cost, highly absorbing materials - such as graphite and metals. Resultant properties of these fluids will be modeled in this section. As a first step in determining optical properties of these nanofluids, we must find the optical properties of the bulk materials used to create the nanofluid. That is, we need to know the complex refractive index (or dielectric constant) of the base fluid and of the bulk nanoparticle material. These can be found for many pure substances in an optical properties handbook, such as Palik [29]. Given this information, it is usually possible to calculate the optical properties of the nanofluid mixture. However, this can be very difficult if the nanofluid is a strongly scattering medium. At higher particle concentrations (typically more than 0.6 vol.%), dependent and multiple scattering phenomena can play a role since the particles are closely packed [30]. However, it turns out for any solar collection with sizable absorption path lengths (anything thicker than 1 mm), an effective solar collector can be achieved at very low volume fractions. Figure 1 is a scattering regime map which helps visualize how 'solar nanofluids' compare to other common fluids. (The figure is modified from Tien [30].) Note that the particle size parameter, *α*, in Figure 1 is defined as [30]:(2)

where *D *is the diameter of the nanoparticle and *λ *is the wavelength of incident light (note: *D *and *λ *must be of the same units to get a non-dimensional *α*). Thus, very small particle sizes and volume fractions make it is safe to assume that we are working in the independent scattering regime which requires relatively simplistic optical properties calculations. Commonly used nanoparticles are in the range 10 to 50 nm of average particle diameter, for which most of the incident light from the sun has a wavelength that is at least ten times larger. This allows one to ignore many of the higher order components found in Mie scattering theory [31]. As a result, the following equations can be used to solve for the scattering (*Q*_scat_), absorption (*Q*_abs_), and extinction (*Q*_ext_) efficiencies, respectively, of individual particles. (These equations are found in several standard texts, such as Bohren and Huffman [32].)(3)(4)(5)

where *m *is the relative complex refractive index of the nanofluid and *α *is the size parameter, which depends on the particle diameter, *D*, and the incident wavelength, *λ *[31].

In nanofluids *Q*_scat _is generally at least an order of magnitude smaller than *Q*_abs _due to the fact that scattering is proportional to *D*^4^. Consequently, scattering is usually negligibly small. However, this is only true if the particles are uniformly small. In reality, some fraction of the fluid may consist of larger particle agglomerates. If it is negligible, the scattering coefficient simply drops out of the following equation for the nanoparticles' extinction coefficient, *σ*_particles _[32]:(6)

Lastly, we must also incorporate any absorption of the base fluid. The approach of Equations 3 to 6 assumes that the base fluid is totally transparent. However, water very strongly absorbs near infrared and infrared radiation. For wavelengths ≥0.9 μm, where approximately 35% of the sun's power is located, water is actually a much better absorber than the nanoparticle materials used in this study. Thus, as a first-order approximation, we propose that the total nanofluid extinction coefficient is a simple addition of the base fluid extinction coefficient, *σ*_basefluid_, and that of the particles, *σ*_particles_. We define these as the following:(7)(8)

Note that *k*_basefluid _is the complex component of the refractive index for the base fluid. Also, for comparison with other research, we choose to present extinction coefficients in cm^-1^. This means that *λ *and the fluid depth, *L*, must be in cm in the following equation of Beer's law [32]:(9)

### Effective medium approach to optical properties

A common approach to modeling properties in a composite material is the Maxwell-Garnett theory. As such, we will attempt to use a Maxwell-Garnett effective medium calculation to calculate the complex refractive index. Equation 10 shows this approach, where the subscripts eff, f, and p define the effective medium (i.e., the nanofluid), the base fluid, and the particles, respectively [32]:(10)

One should note if *ε*_f _is very small, as it is in the complex dielectric component for water (from 0.1 to 1 μm), large rounding errors may occur when using this approach. This limits the applicability of this method. Once the effective dielectric constant is found, it is relatively easy to convert back to the refractive index using [32]:(11)(12)

**Figure 2:**
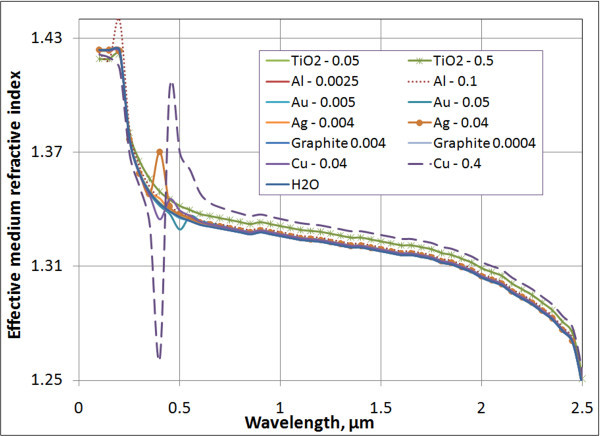
**Maxwell-Garnett approximation of the real part of the refractive index for water-based nanofluids**. The numbers in the legend represent the volume fractions of the specified nanofluids with 30 nm of average particle size.

In Equations 11 and 12, *ε*' and *ε*" represent the real and imaginary components of the dielectric constant. The real part, *n*_eff_, of the refractive index for several nanofluids, determined from Equations 11 and 12, is plotted in Figure 2. Since there is, at most, a factor of ten difference (and in many cases less than 100% change) in the real part of the refractive index between the bulk particle material and the base fluid, this approach gives rather accurate results. Figure 2 shows little deviation from the real part of the refractive index for low volume fractions, which is logical. Note: Properties for the bulk materials were taken from Palik [29] for the effective medium analysis.

**Figure 3:**
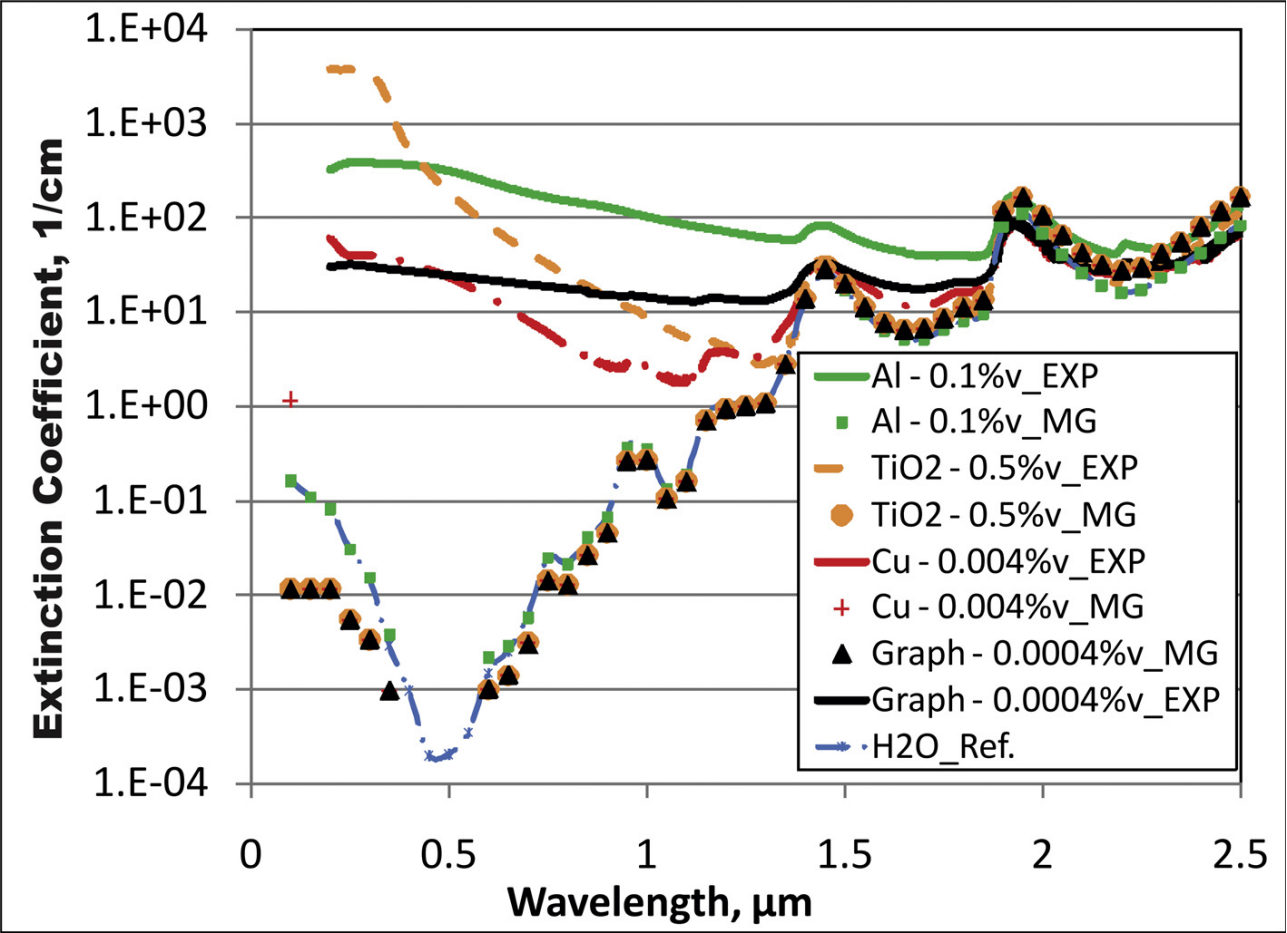
**Maxwell-Garnett modeling of the extinction coefficient for water-based nanofluids**. Where "MG" is the calculated value based on the Maxwell-Garnett model (Equation 10) and "EXP" are measured values.

For the imaginary component, *k*_eff_, the effective medium approach yields a severe underprediction. For the sake of consistency, Figure 3 also plots extinction coefficients, which are calculated using Equation 7, with *k*_eff _replacing *k*_basefluid_. The results given in Figure 3 are many orders of magnitude below the measured values for these volume fractions. In the visible range, *k*_eff _for water is many orders of magnitude (approximately ten) less than that of metal nanoparticles. Due to this large difference, the Maxwell-Garnett theory is generally not an accurate approach to obtain the extinction coefficient for nanofluids.

### Scattering issues

It should be noted that the extinction coefficient is composed of the absorption coefficient and the scattering coefficient. If particles are nano-sized and far apart, the scattering component of the absorption coefficient will be small compared with the absorption component - but not zero. One major failing in modeling optical properties is assuming the size of the particles to nominally be that of quoted by the manufacturer. In general, this is not true since the particles always agglomerate to some extent with the two-step method of preparation. Dynamic light scattering results indicate the real average particle diameter to be 50 to 120 nm, instead of the manufacturer-quoted 20 to 40 nm. This can significantly change the amount of scattering that occurs in a nanofluid. Equation 13 presents a simplified relationship for finding the fraction of incident light that is scattered [32]:(13)

where *D *is the particle diameter, *N *the number of scattering particles in the beam path, *λ *the wavelength of light, *m *the relative complex refractive index, and *θ *the scattering angle. Thus, a tripling of the diameter (from 30 to 90 nm) gives a 730-fold increase in the amount of scattering! Thus, if particles in a real nanofluid are larger than what is assumed above, scattering may cause deviations from the model.

## Experimental approach

Creating a stable nanofluid is a must for any real application and for measuring optical properties. Without careful preparation, nanoparticles will agglomerate and settle out of the base fluid in a very short time. Although there are many methods of nanofluid preparation, they can be roughly categorized into "one-step" and "two-step" processes. One-step processes synthesize the nanofluid to the desired volume fraction and particle size inside the base fluid. Thus, the final product is a specific nanofluid which is ready for use (possibly after dilution). The two-step method is accomplished by first synthesizing the dry nanoparticles to a preferred size and shape. In the second step, these particles are carefully mixed into the desired base fluid at the desired volume fraction, usually with some additives for stability.

Several researchers have had success fabricating and testing nanofluids using one-step preparation methods [33-35]. Based on these results, one-step methods may produce the best results for commercial applications if they can be scaled up and manufactured inexpensively. However, due to its straightforward nature and its controllability, we will only use and discuss the two-step method.

A variety of dry powders are available "off-the-shelf" [36-38]. These particles can be mixed into many different liquids at the preferred concentration. Depending on the stability and quality required, this process can take anywhere from a few minutes to several hours. For the test fluids of this article, the particles and up to 1% sodium dodecyl sulfate (a surfactant) were dispersed into the base fluid using a sonicator (a UP200 from Hielscher Ultrasonics GmbH, Teltow, Germany) for 15 to 30 min. From our experience, probe-type sonicators break particle agglomerates faster and much more thoroughly than bath-type sonicators. Since it is relatively quick, requires very little "high tech" equipment, and produces any number of nanofluids, this process is our method of choice. Unfortunately, surfactant-stabilized nanofluids are known to break down at elevated temperature [39]. For longer-term stability in a solar application, one can re-sonicate continuously or attempt more exotic preparation methods, such as those given in [34,40].

To measure the optical properties, we used a spectrophotometer. This is a device that sends a light beam of variable wavelength through a sample and then detects the transmitted beam. Spectrophotometers come in several configurations and are good for a variety of wavelengths. For our purposes, we need measurements over the solar spectrum, i.e., between 0.20 to 3 μm. As such, we mostly use a Jasco V-670 (Jasco Corp., Great Dunmow, Essex, UK) which can take transmission measurements in the range of 0.19 to 2.7 μm, although other spectrophotometers are used for comparison in our testing.

**Figure 4:**
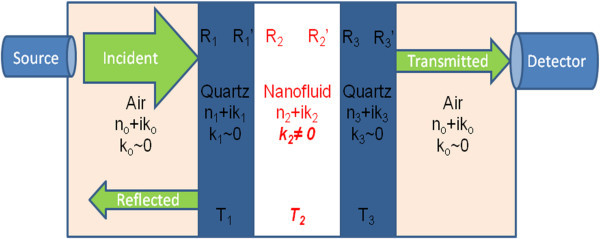
**Diagram of the three-slab system representation for a spectrometry measurement of a nanofluid-filled quartz cuvette**.

Regardless of the spectrophotometer used, some further calculations are necessary to obtain extinction coefficients for nanofluids. Since a cuvette contains the liquid sample in the system, the resulting measurement is actually that of a 'three-slab system'. This adds complexity since there can be multiple reflections at each interface which needs to be taken into account in the measurements. Figure 4 shows the details of this multi-component system.

As can be seen in Figure 4, some of the signal going through the three-slab system is lost to reflections at the interfaces. With known refractive indices of quartz and air, it is possible to determine the nanofluid optical properties. As a first step, we calculate values of reflection *R *and transmission *T *shown in Figure 4 in accordance with the approach of Large et al. [41]:(14)(15)

The variables *n_i _*and *k_i _*in the previous equations represent the *i*th spectral real and imaginary components of the refractive index. Likewise, *L *represents the length of the *i*th element. To combine these equations for a two-element system, the following equations can be used [41]:(16)(17)(18)

Following the same process, a further combination for three elements can be done with the following formula [41]:(19)

With these defined, an iterative calculation of the complex index of refraction is possible. Using the imaginary part of the nanofluid index of refraction, *k*_EXP_, a simple calculation can be performed to obtain the extinction coefficient, *σ*_exp_. Equation 20 describes this final step [31]:(20)

If our simplistic nanofluid model is accurate, *σ*_EXP _should be directly comparable to the modeled quantity, *σ*_total_, described in the previous section.

To determine the particle size in solution, dynamic light scattering (DLS) was done for selected materials - graphite (30 nm manufacturer-quoted average particle size (APS)) and silver (20 nm manufacturer APS). The equipment used to do these measurements was a Nicomp 380 DLS (Agilent Technologies, Inc., Santa Clara, CA, USA). Results gave volume-weighted average particle sizes to be 150 to 160 nm and 50 to 70 nm for graphite and silver, respectively. In both cases, the standard deviation was around half of the volume-weighted average. DLS testing also revealed that 24 h later the samples heavily clumped into 1 to 15 μm aggregates, showing that our preparation method for these fluids is only good for short-term stability. It should be noted that the volume-weighted average yields particle sizes that lie between number and intensity-weighted averages.

## Results and discussion

**Figure 5:**
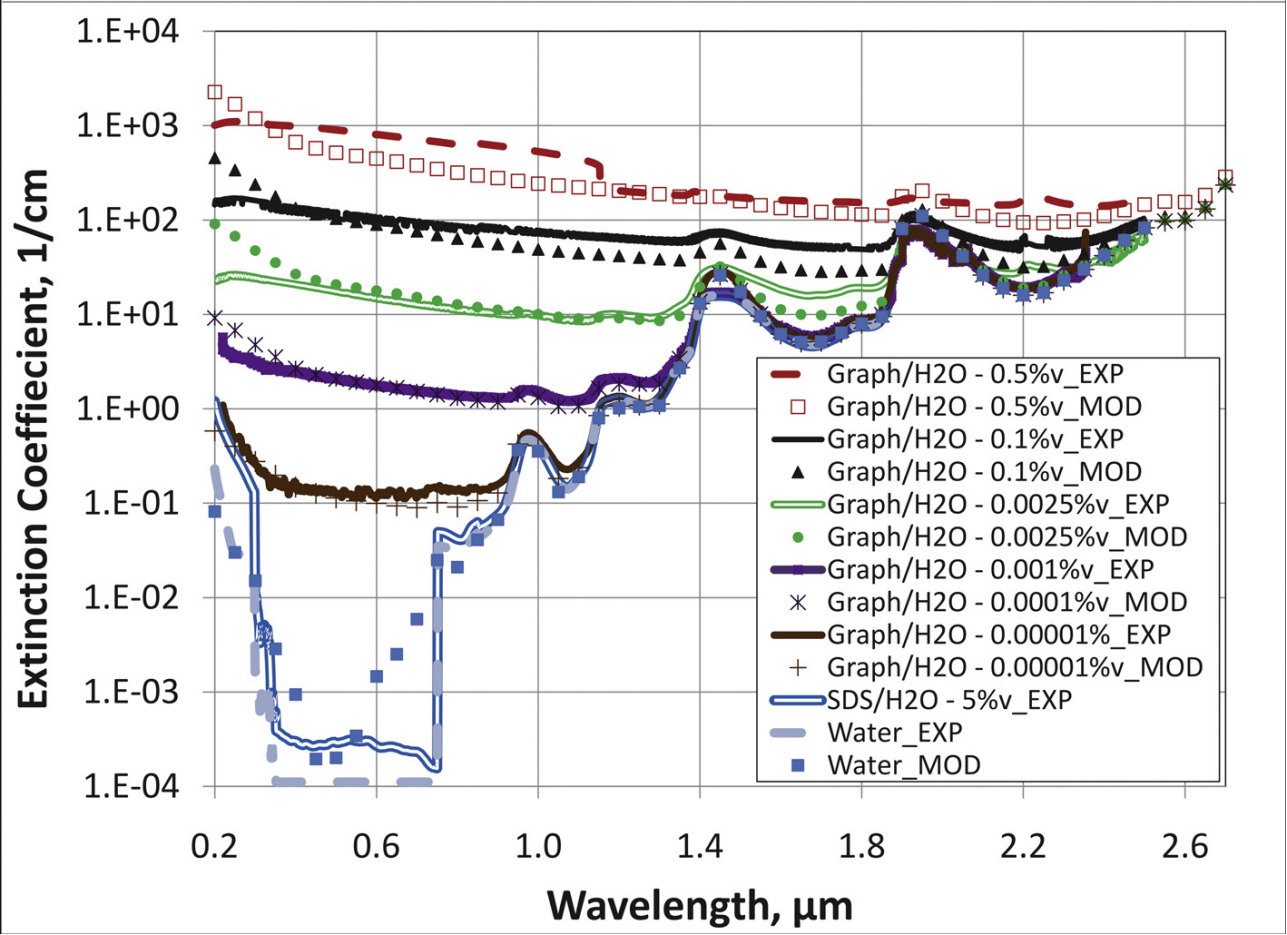
**Modeled and experimental extinction coefficients for several concentrations of aqueous graphite nanofluids**. Experimental results for pure water and water with 5 % surfactant are also plotted for comparison.

To compare the approaches discussed above, Figure 5 shows several concentrations of water-based graphite nanofluids - nominally 30 nm in diameter of spherical particles. Experimental (labeled "EXP") and modeling (labeled "MOD") results are plotted together. Due to the large number of data points, the measured/experimental results are shown as lines while the modeling results are shown as marker curves. Note that the curve labeled "Water_MOD" is essentially data from the reference book by Palik [29]. That is, Equation 16 is used to manipulate reference text data from the complex refractive index, *k*_EXP_, to the extinction coefficients shown in the plot. For comparison, pure water with an excessive amount, 5% by volume, of surfactant is also shown. A high volume fraction surfactant was used to exaggerate the absorption of surfactant, which turns out to be very small.

The concentrations shown in Figure 5 represent a very wide range which could accommodate almost any solar receiver geometry. Overall, there is very good agreement between model and experimental results. Depending on volume fraction, the nanoparticles appear to be the absorbing material for shorter wavelengths (up to approximately 1 μm for 1 × 10^-5 ^vol.% and up to approximately 2 μm for 0.1 vol.%), whereas at longer wavelengths, water becomes dominant and the curves converge. These results indicate that our simplistic approach (i.e., Equations 2 to 9) agrees well with experimental data.

**Figure 6:**
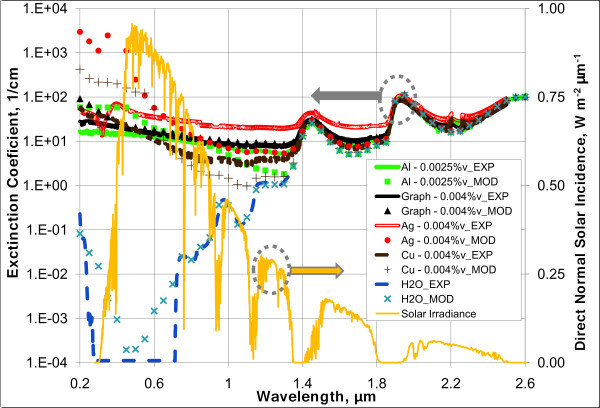
**Extinction coefficients - measurements versus modeling for promising water-based "solar nanofluids"**. The curve which is the lowest on the right part of the graph represents the irradiance directly hitting a normal surface for a mid-latitude summer location in the United States.

Conventional solar receivers have fluid depths on the order of 10 cm. Thus, a real nanofluid solar receiver would likely have a similar geometry. Figure 6 shows some characteristic results for several water-based nanofluids which were chosen to absorb > 95% of incoming solar radiation over this fluid depth. Direct normal solar irradiance is also shown over the same wavelengths for comparison in Figure 6. Again, one can see the characteristic high extinction coefficients for the nanoparticles at short wavelength and that of water at longer wavelengths, ≥1.1 μm. For this fluid thickness, the nanoparticles will be absorbing approximately 65% to 70% of the incoming solar energy, with the base fluid, water, absorbing approximately 30%.

Since the base fluid is a good absorber at longer wavelengths, it will also be a good emitter at those same wavelengths. That is, most nanofluids are also expected to have radiation losses nearing those of a blackbody at longer wavelengths (> 4 μm) according to Plank's radiation law. There are two possible solutions to this problem for a solar collector: (1) find a base fluid which has low emission for long wavelengths and (2) install a cover/glazing over the collector which will trap long-wavelength emitted radiation from leaving the system. The second solution is most likely to be adopted since (as mentioned above) there are many commercial materials which could be used to minimize losses and are still essentially transparent to the solar spectrum [26,27].

Figure 6 also shows less agreement between the model results and the experimental results for metals than is seen for graphite. Most noticeably in silver, we expected to see a large peak in the extinction coefficient. This peak, referred to as the plasmon peak, is a built-in natural frequency where electrons will absorb and oscillate strongly in a metal. It is usually found in the range of 200 to 500 nm. However, our experimental results for metal-based nanofluid were rather constant and did not show a large, pronounced plasmon peak as expected. In general, our model for metal nanofluids appears to over-predict from very short wavelengths until around 600 to 700 nm where it then begins to under-predict the extinction coefficient.

**Figure 7:**
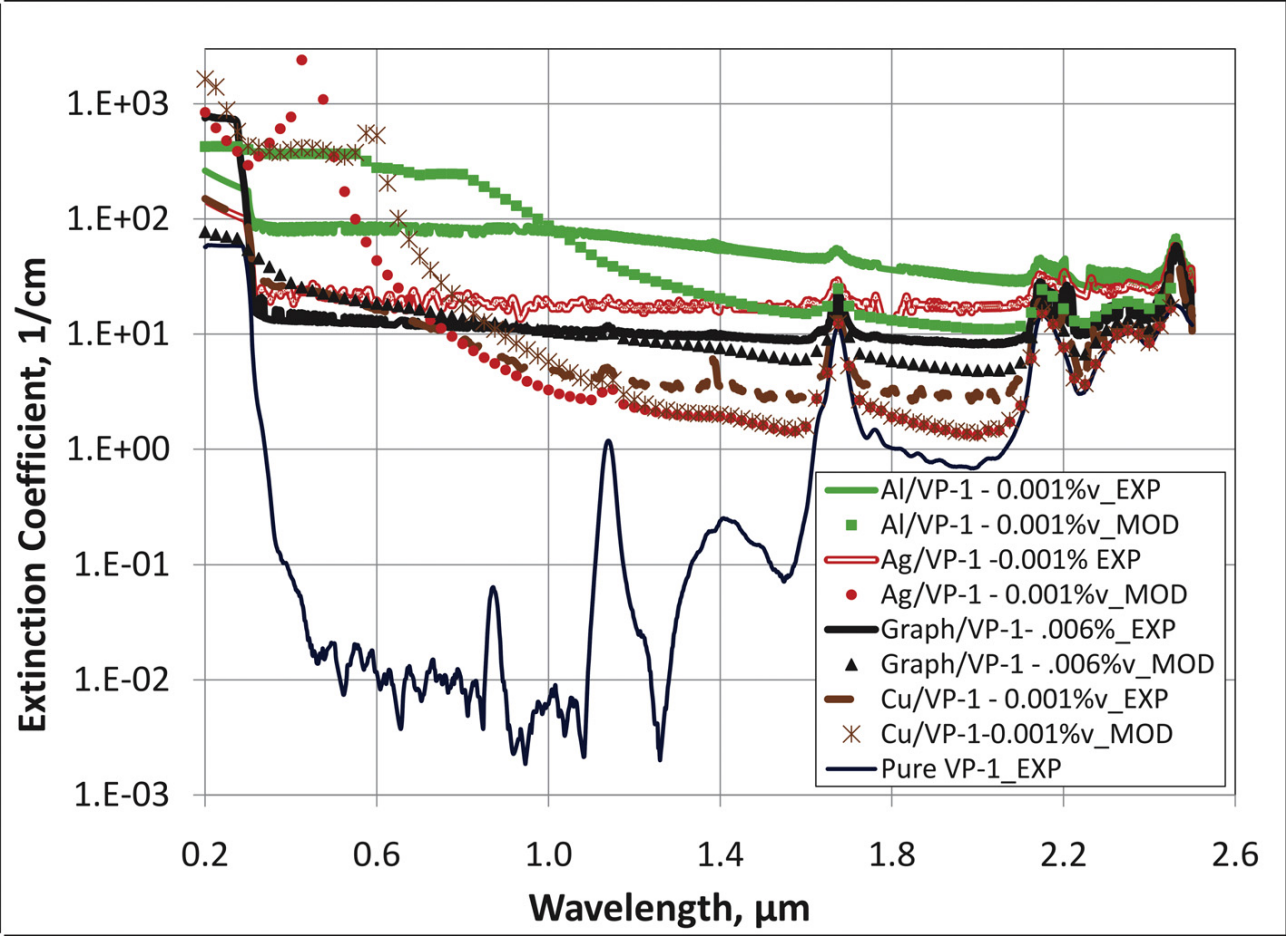
**Extinction coefficients for Therminol VP-1-based "solar nanofluids"**. Bottom curve shows experimental results for the pure base fluid, Therminol VP-1.

Figure 7 shows similar plots for various nanofluids which have Therminol VP-1 (Solutia Inc, St. Louis, MO, USA) as a base fluid. Therminol VP-1 is a type of heat transfer fluid which is commonly used in many solar collectors. It is a colorless liquid which is only slightly more viscous than water and has a much higher boiling point, approximately 257°C. This ability to work at higher temperature makes it applicable for medium-temperature solar collectors. It is composed of approximately 26.5% biphenyl and 73.5% diphenyl oxide. Unfortunately, there is very little information on the optical properties of these materials. Thus, the experimentally determined properties for the base fluid are used in the modeled extinction coefficients in Figure 7. Very similar trends are present to those seen in Figure 6, except that the absorption of the base fluid is less dominant at longer wavelengths.

The accuracy of this system is at least ± 0.3%*T*. Thus, if we get a result of 90% transmission, it could actually be 89.7% or 90.3% transmission. However, the poor match in results in Figures 6 and 7 cannot be explained by this error. One possible reason for the discrepancy, however, is that particle agglomerates are in the measurement beam path and absorb or scatter an anomalously large amount of light. That is, the real particle *shape *or *size *might deviate from the nominal manufacturer-stated nanoparticle specifications. Furthermore, the model assumes a monatomic particle distribution. That is, all the particles of a given sample are assumed to be the same size - thus, the average particle diameter quoted by the manufacturer. Another possible explanation for the poor agreement is that an oxide layer or other chemical deviation may occur in the metal nanoparticles giving different properties than that assumed in the bulk metal.

**Figure 8:**
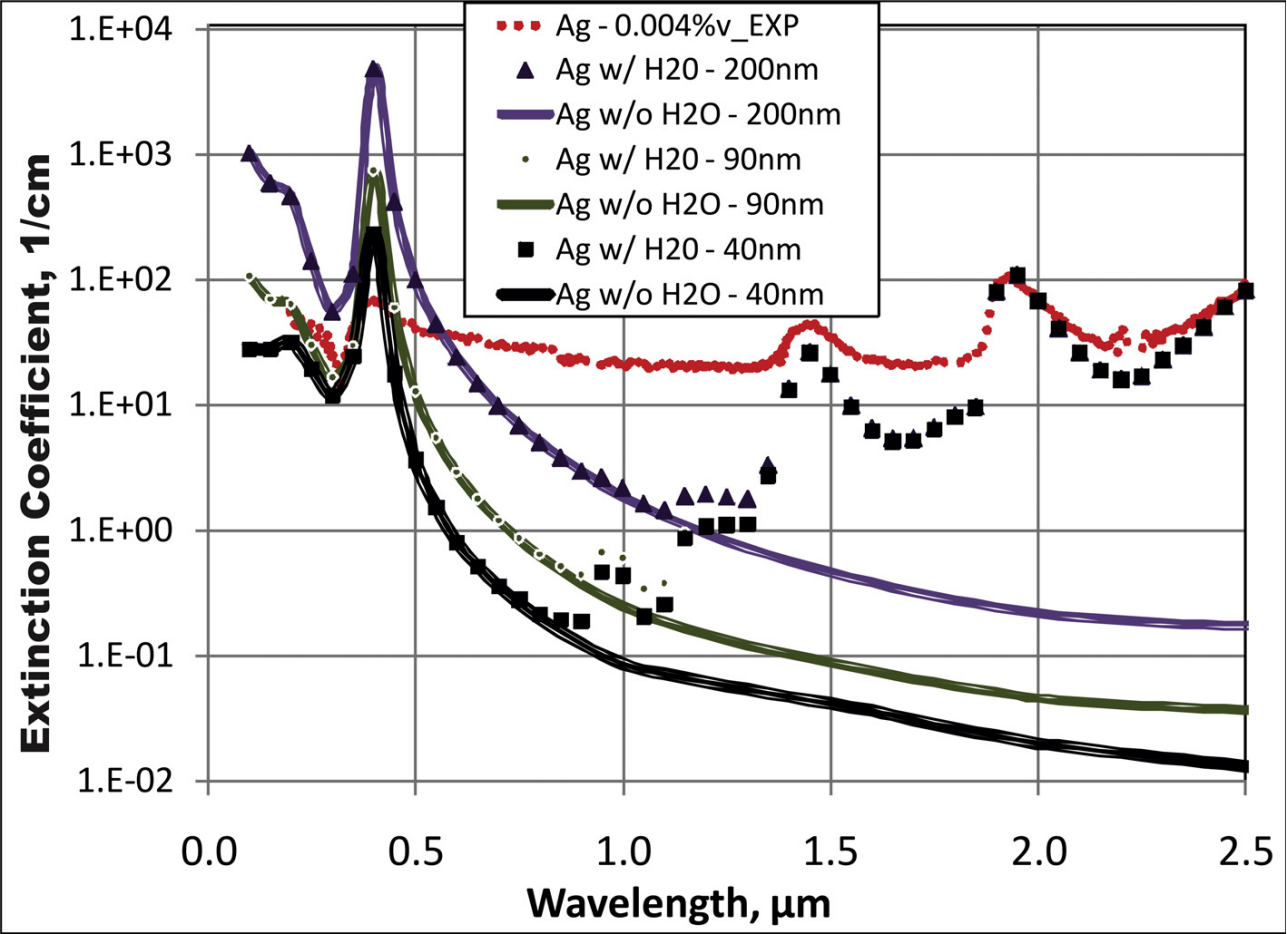
**Extinction for different particle diameters and the absorption of water in a 0.004-vol.% silver nanofluid**. "EXP" = experimental results for silver with manufacturer-quoted 40 nm of average particle size.

Particle size can be adjusted in our model. As a first check, we can explore this as the possible root of the problem. Since silver nanofluid shows the most deviation between model and experimental findings, we should look into the effect of varying particle size in silver nanofluids. Extinction coefficients of several 0.004% volume fraction silver nanofluids with a variety of nominal particle diameters are plotted in Figure 8. The experimental result for this volume fraction of particles with a manufacturer-quoted average particle size of 40 nm is also shown for comparison to the various model plots. Further, curves for *σ*_total _and *σ*_particles _are plotted together to demonstrate the effect of absorption by the base fluid. This shows the importance of adding in the extinction of the base fluid into the total result. Overall, Figure 8 shows that size effects, while very important, do not seem to explain the difference between the rather flat trend of the experimental results and the large peak in the theoretical model.

As mentioned above, scattering can also come into play, especially important at short wavelengths. Taking the results of Figure 8 and a nominal particle size of 100 nm, up to 5% of the incident light can be scattered in a solar nanofluid. In a 10-cm fluid depth, this translates to an average extinction coefficient of 0.05 cm^-1^. Overall, these results show that a measurable amount of light can be scattered if large particles or particle agglomerates are present. If the particle size is < 50 nm, however, scattering is negligible - so care must be taken to make sure that the particles in a nanofluid stay "nano."

## Conclusions and future work

This article has shown measurement and modeling techniques for determining the optical properties of nanofluids. These two methods of determining optical properties are in very good agreement for graphite nanofluids. They also correspond well in the case of aluminum. However, experimental results did not match well with the model predictions for the other metals tested, particularly missing the large predicted plasmon peaks (e.g., silver). Particle size was discredited as the root of poor model predictions for metals. Scattering is expected to be negligible if care is taken to keep particles in solution near their manufacturer-listed diameters - so this is also unlikely to lead to significant errors. One possible explanation is purity of the materials. For instance, oxidization or other impurities on the particle surface might be responsible for the poor agreement with the model.

For modeling extinction coefficients in absorbing materials, the Maxwell-Garnett effective medium approach does not appear to correctly predict the extinction coefficient for nanofluids. The main drive of this research was to find nanofluids which make effective direct absorption solar collection media. As such, the results of this article can be used to provide some guidance to those looking to build (or retrofit) a nanofluid-based direct absorption solar collector. Table 1 gives a list of recipes for making these nanofluids with the two-step method. Each nanofluid shown in Table 1 is expected to absorb > 95% of the AM1.5 direct normal radiation for a 10-cm fluid depth. It should be noted that the desired operational conditions, solar concentration ratio, and the collector geometry/construction will affect the overall receiver efficiency. The table indicates that graphite and aluminum nanofluids provide very good value. Graphite and/or aluminum nanofluids (which can be relatively accurately predicted) are more likely to find their way into real direct absorption solar collectors due to the significant price difference in the raw materials. This article also indicates that absorption is mostly due to the nanoparticles at shorter wavelengths and mostly due to the base fluid at longer wavelengths. Thus, it is reasonable to approximate the total extinction coefficient as the sum of the extinction from the particles and that of the base fluid as given in Equations 2 to 8.

**Table 1:** Solar thermal nanofluid comparison table

Type	Graphite	Al	Copper	Silver	Gold
Particle, vol.%	0.0004	0.001	0.004	0.004	0.004
Commercially available	Yes	Yes	Yes	Yes	Yes
Surfactant, vol.%	0.5	0.25	0.25	0.25	0.25
1M NaOH, vol.% (achieve pH 9 to 10)	0.003	0.003	0.003	0.003	0.003
Sonication time, min	45	30	30	30	30
Collector depth, cm	10	10	10	10	10
Approximate cost, $/L	0.52	0.64	1.85	3.65	233

Assumes pure water base - where water + stabilizers = $0.5/L).

Further work will be necessary to obtain better models for nanofluids containing metallic nanoparticles other than aluminum. Also, a more in-depth study will be required to obtain optical properties at elevated temperatures. Since liquid-based solar thermal collectors can operate anywhere from 50°C to 500°C, it is very important to characterize these properties at those temperatures. We predict that nanofluids would be most cost-effectively placed into solar systems with a relatively small receiver area (such as a power tower or dish receiver), but more work must be done to determine the most advantageous use of solar nanofluids.

## Abbreviations

### NOMENCLATURE

*D*: Mean particle diameter (nm); *f*_v_: Volume fraction (%); *I*: Irradiance, W m^-2^; *k*: Complex component of the refractive index; L: Path length, mm; *m*: Relative complex refractive index (particles to fluid); *N*: Number of scatterers; *n*: Real component of the refractive index; *Q*: Optical efficiency factor; *R*: Reflectivity; *T*: Transmissivity.

#### Subscripts

║: Parallel component; ┴: Perpendicular component; abs: Absorption; e: Effective; ext: Extinction; EXP: Experimental result; F: Fluid; MOD: Modeling result; scat: Scattering.

#### Greek symbols

*α*: Particle size parameter; *ε'*: Real component of the dielectric constant, F/m or (kg mm mV^-2 ^s^-2^); *ε"*: Complex component of the dielectric constant, F/m or (kg mm mV^-2 ^s^-2^); *θ*: Scattering angle, radians; *λ*: Wavelength, μm; π: The constant, pi; *ρ*: Density, kg/m^3 ^or #/m^3^; *σ*: Extinction coefficient, 1/cm.

## Competing interests

The authors declare that they have no competing interests.

## Authors' contributions

RT led the effort by conducted the experiments and preparing the manuscript. PP helped design the experimental study and advised on the project. TO developed the modeling techniques and wrote most of the numerical coding. RA helped design the experimental and modeling study and advised on the entire project. All authors read, edited, and approved of the final manuscript.
